# The *Mycobacterium tuberculosis* PE_PGRS Protein Family Acts as an Immunological Decoy to Subvert Host Immune Response

**DOI:** 10.3390/ijms23010525

**Published:** 2022-01-04

**Authors:** Tarina Sharma, Anwar Alam, Aquib Ehtram, Anshu Rani, Sonam Grover, Nasreen Z. Ehtesham, Seyed E. Hasnain

**Affiliations:** 1 ICMR-National Institute of Pathology, Ansari Nagar West, New Delhi 110029, India; tarina.sharma2@gmail.com (T.S.); dranwar.iit@gmail.com (A.A.); anshuranibiotech@gmail.com (A.R.); 2 Kusuma School of Biological Sciences, Indian Institute of Technology Delhi, New Delhi 110016, India; ehtramaquib@gmail.com; 3 Jamia Hamdard Institute of Molecular Medicine, Jamia Hamdard, Hamdard Nagar, New Delhi 110062, India; sonamgbt@gmail.com; 4 Department of Biochemical Engineering and Biotechnology, Indian Institute of Technology Delhi (IIT-D), Hauz Khas, New Delhi 110016, India; 5 Department of Life Science, School of Basic Sciences and Research, Sharda University, Knowledge Park III, Greater Noida, Uttar Pradesh 201310, India

**Keywords:** decoy antigens, glycine, immune evasion, latency, pathogenicity, TB, virulence

## Abstract

*Mycobacterium tuberculosis* (*M.tb*) is a successful pathogen that can reside within the alveolar macrophages of the host and can survive in a latent stage. The pathogen has evolved and developed multiple strategies to resist the host immune responses. *M.tb* escapes from host macrophage through evasion or subversion of immune effector functions. *M.tb* genome codes for PE/PPE/PE_PGRS proteins, which are intrinsically disordered, redundant and antigenic in nature. These proteins perform multiple functions that intensify the virulence competence of *M.tb* majorly by modulating immune responses, thereby affecting immune mediated clearance of the pathogen. The highly repetitive, redundant and antigenic nature of PE/PPE/PE_PGRS proteins provide a critical edge over other *M.tb* proteins in terms of imparting a higher level of virulence and also as a decoy molecule that masks the effect of effector molecules, thereby modulating immuno-surveillance. An understanding of how these proteins subvert the host immunological machinery may add to the current knowledge about *M.tb* virulence and pathogenesis. This can help in redirecting our strategies for tackling *M.tb* infections.

## 1. Introduction

Tuberculosis (TB), caused by the opportunistic pathogen *Mycobacterium tuberculosis* (*M.tb)*, is a deadly disease and a major cause of death globally, [1] The prognosis of TB is further worsened due to co-morbid conditions, such as infections of HIV, and now the ongoing COVID-19 pandemic is posing additional challenges [2]. The emergence of drug resistant forms (MDR and XDR) of *M.tb* is a cause of concern as it has slowed our efforts to eradicate TB worldwide [3]. Macrophages are primarily efficient in clearing pathogens; however, *M.tb* can survive intracellularly within the niche of macrophage itself. *M.tb* has evolved various mechanisms that allow it to hijack the process of phagosome-lysosome fusion, inhibit acidification of phagosome, suppress autophagy and apoptosis pathways used by macrophage for the clearance of pathogens [4,5,6,7]. *M.tb* overpowers the extremely microbicidal nature presented within the macrophages through a multifaceted and complex interplay between its proteins and host immune responses [8,9]. Several *M.tb* proteins have been reported to evoke innate and adaptive immune responses, though many of these act as decoy antigens to subvert the immune system. Decoy antigens mimic host-pathogen effector components and can misdirect the immune response pathways that favor the survival of the pathogen. Pathogenic bacteria such as *M.tb* also use decoy proteins as a generic mechanism to mask themselves from immune surveillance, thereby evading and subverting host immune responses [10,11]. The decoy antigens can be classified into three broad categories, namely, receptor, bodyguard and sensing decoys. As the name suggests, receptor decoys are employed by the pathogens to modulate host immune signaling pathways. In contrast, bodyguard decoys act as inactive mimics to safeguard the virulence factors of pathogens from the host response. Sensing decoys mimic the effector functions of the target proteins of both the host and the pathogens [12].

The mycobacterial PE/PPE/PE_PGRS protein family, present only in the genus mycobacterium and nowhere else in the living kingdom, occupies approximately 10% of the coding capacity of the *M.tb* genome. Despite the reductive genomic evolution of *M.tb* [13], the PE/PPE/PE_PGRS family of genes has been expanding during mycobacterial evolution. The presence of this family only in pathogenic strains of the genus mycobacterium, such as *M.tb, M.marinum* and *M.bovis*, points to its likely importance in disease pathogenesis [14]. The evolution of PE/PPE gene families was found to be associated with the ESX secretion system [15], and proteins were majorly reported to be either surface exposed or secreted [16,17,18]. Cell-surface localization of PE/PPE/PE_PGRS proteins may serve an important function in host-pathogen interactions and in the virulence and pathogenesis of *M.tb* [19]. PE_PGRS (polymorphic GC-rich sequences) proteins are a subclass of the PE protein family and consists of a highly conserved N-terminal (approx. 110 amino acid long) the PE domain followed by the C-terminal domain harboring multiple repeats of Gly-Gly-Ala or Gly-Gly-Asn. Deciphering the role of proteins belonging to the PE_PGRS family may reveal new aspects of the biology of *M.tb*. The presence of multiple tandem repeats of GGA or GGN has been attributed to cause antigenic variations and aid in immune evasion mechanisms, thereby facilitating pathogen survival. The repetitive nature of PE_PGRS proteins and their surface localization both lead to the generation of immune responses by macrophages and may aid in immune subversion [17].

In this review, we highlight the various hypothesis and published data to showcase the role of antigenic *M.tb* PE/PPE/PE_PGRS proteins in evading and subverting host immune responses, which in turn favor *M.tb* survival and dissemination. It is envisaged that a better understanding of PE_PGRS proteins shall provide crucial insights about *M.tb* virulence and immune evasion and may aid in designing alternative approaches to design better vaccines and therapeutics.

## 2. Modulators of the Host-Immune Response: PE/PPE Proteins

PE_PGRS and PPE-MPTR (major polymorphic tandem repeat) make up the majority of the PE/PPE proteins, which are associated with cell wall and are secretory in nature [20,21]. These proteins play an important role in the development of mycobacterial pathogenicity by modulating the host immune system. PE and PPE protein are implicated in the manipulation and evasion of the host immune system. *M.tb* primarily infects the macrophages, which act as a reservoir for the pathogen. The expression pattern of these proteins is significantly altered in *M.tb* when it infects the macrophage, pointing to their role in virulence [22]. *M.tb* uses a variety of ways to counteract macrophage-mediated protective mechanisms [23]. *M.tb* exploits an extra lipoidal cell wall with the presence of PDIM (phthiocerol dimycocerosates) to protect the pathogen from the host’s innate immune surveillance. Before the adaptive immune response kicks in, *M.tb* establishes itself in the host macrophages [24,25]. A number of PE/PPE proteins influence macrophage activity by modulating or changing the cellular dynamics. *M.tb* pathogenicity is influenced by the Esx5 secretion system and its substrates, which include associated and non-associated PE/PPE proteins.

Although there is evidence that PE/PPE proteins have a variety of roles in modifying the host intracellular milieu during infection, more research is necessary to understand this. Many PE/PPE proteins control the host immune response by upregulating the production of cytokines that trigger pro-inflammation or anti-inflammation [23,26,27,28]. PPE37, for example, modulates the pro-inflammatory response in the host by lowering the production of IL-1β, IL-6, and TNF-α [29]. PE5- and PE15-expressing *M. smegmatis* recombinants increased the intracellular bacterial survival in the host, suggesting that they may play a role in the host-pathogen interaction. Infected macrophages showed up- and down-regulation of IL-4, IL-5, IL-10, and TGF-β (anti-inflammatory cytokines), and IL-12 (pro-inflammatory cytokine). PE5, PPE15 and PPE2 inhibit reactive nitrogen species’ production and are predicted to be an anti-mycobacterial strategy of the host [30,31].

T-lymphocytes are important contributors in the secretion of the cytokines. A critical balance between the Th-1 and Th-2 subsets of the T-lymphocytes drives the response against varied infections. Th1 response is critical in wiping out the intracellular pathogens whereas Th-2 is essential in clearing the extracellular infections. Th1 polarization is inclined towards the secretion of the pro-inflammatory cytokines and thus attracts the cytotoxic cell population towards the infected cells to handle the intracellular pathogens. On the contrary, Th-2 polarization is involved in the clonal expansion of the B-cells which leads to an increase in the antibody titres in the system that act on the extracellular pathogens. Th2 cells also secrete anti-inflammatory cytokines such as IL-1, IL-4 and IL-10, etc. During inflammation, due to mycobacterial infection, Th-1 polarization is important to clear the infection from the infected tissues. However, it should be balanced by the Th-2 response to control the Th-1 mediated excessive tissue damage. Consequently, to counter host defense, several PE/PPE proteins, such as PE4, PE5, and PE15, disrupt Th-1 and Th-2 equilibrium, bypassing the host immune response and allowing bacteria to survive longer inside macrophages [30,32]. The sera of the TB patients generated a substantial antibody response when probed against the antigen PPE41 [33,34]. The co-translated PE32/PPE65 heterodimer has recently been shown to influence host immune response in favor of the pathogen by dampening the Th-1 response [35]. The Esx5 associated PE paralogs PE18 and PE19 twisted the equilibrium towards Th1 bias [36]. PE/PPE proteins that drive the Th-1 response might play a role in diverting the immune attention away from the major virulent effectors of *M.tb.*

PE13, which establishes cell wall function, was also discovered to be implicated in the interplay between pathogen and host signaling, via the p38-ERK-NF-κB axis. It induces macrophage apoptosis and subsequent cell-to-cell dispersion, resulting in increased mycobacterial survival in macrophages over time. PE13 boosted macrophage secretion of IL-1 and IL-6 and decreased the suppressor of cytokine signaling 3 (SOCS3) synthesis [37]. PPE32, such as PE13, promotes mycobacterial intraphagocyte survival through an aberrant increase in cytokine production, particularly IL-12 and IL-32 [38]. ESAT-6 and PPE25, proteins implicated in both bacterial pathogenicity and host immune recognition, were found to interact in *M. avium*. PPE25 was shown to be localized to the bacterial cell membrane after being expressed in *M. smegmatis* [39]. The localization of PPE25 in the cell membrane suggests that it would install a direct contact with the host’s immune system during infection.

PE11, also known as LipX, is shown to be elevated in active tuberculosis patients and is restricted to the pathogenic mycobacterial species. It promotes virulence by changing the structure, composition, or alignment of the outer cell wall [40]. PE_PGRS62, PE_PGRS30, and PPE25 (homolog from a subspecies of *M. avium*) have also been linked to mycobacterial virulence and have been shown to hinder phagosome maturation [41]. The *ppe38* deletion mutant of *M. marinum* modified the bacterial cell surface properties, lowering pathogenicity via reducing macrophage phagocytic functions [42]. When the complete *pe/ppe* genetic stretch was removed from the *esx-5* gene locus, the deleted *M.tb* mutant (Δ*ppe25*–*pe19*) showed attenuation in host macrophages as well as in the severe combined immunodeficient mice infection paradigm [20].

Only pathogenic mycobacteria have the ability to infect new uninfected cells, implying that *M.tb* colonizes and multiplies by modulating programmed cell death [43]. We earlier reported that PGRS domain of PE_PGRS5 protein is implicated in TLR4-dependent endoplasmic reticulum (ER) stress-mediated cell death, which may aid pathogen dispersion later in infection [44]. Furthermore, we demonstrated that low cellular iron content causes PPE37 to be proteolytically cleaved, resulting in two segments at the N and C termini, respectively. These segments are responsible for their extracellular and nuclear localization, with the C-terminus inducing apoptosis and the N-terminus modulating the host immune response to favor the pathogen [45]. Furthermore, it was postulated that the PE25/PPE41 heterodimer promotes necrosis in macrophages, rather than apoptosis, to aid in bacterial multiplication and spread, perhaps leading to disease reactivation [46]. However, the significance of apoptosis in *M.tb* infection, disease establishment, and proliferation is yet unknown and hotly debated. Necrotic cell death, on the other hand, is a similar mechanism that facilitates in the spread of bacterial cells from pre-infected macrophages to infect new cells [46]. It is unclear how *M.tb* causes host cell necrosis while also managing its proliferation, especially during the latency phase. To successfully resist the humoral and cell-mediated adaptive immune responses of the host against the established infection, mycobacteria employ necrosis as a cell death mechanism. Dheenadhayalan et al. showed that recombinant *M smegmatis* expressing *M.tb* PE_PGRS33 exhibited increased persistence within the macrophages as compared to the parental strain of *M smegmatis*. A significantly higher number of nucleosomes in the culture supernatant of the macrophages infected with *M smegmatis* expressing PE_PGRS33 pointed to greater cellular destruction. It was found that PE_PGRS 33 altered the levels of TNF secretion in the macrophage, which not only induced necrosis of the cell but also enhanced the survival of the recombinant *M smegmatis* expressing PE_PGRS within the infected macrophages [47]. *M smegmatis* expressing PE_PGRS33 induced necrosis in macrophages similar to that caused by infection with virulent *M tuberculosis*, while *M smegmatis* expressed only that the PE domain failed to induce cell death. This study pointed that the PGRS domain of the PE_PGRS protein can have a specific role in part in inducing necrosis in macrophages.

## 3. Ambiguous Immune Responses of PE/PPE/PE_PGRS Proteins of *M.tb*

Numerous studies focusing on the moonlighting functions of the PE/PPE/PE_PGRS family of proteins revealed their diverse functional implications in *M.tb* pathogenesis during the course of infection. It is of prime importance to note that, while a dozen of these proteins induce pro-inflammatory immune responses, others mount anti-inflammatory responses via separate signaling cascades. The fine-tuning between the two eventually decides the outcome of the disease progression and pathogen survival.

PE/PPE/PE_PGRS proteins serve as possible virulence factors and act as a source of antigenic variation in different *M.tb* clinical strains [17,22]. In silico comparative genomic and proteomic analysis of PE/PPE/PE_PGRS proteins revealed significant differences between their sequences in *M.tb* H_37_Ra and H_37_Rv; that have been translated in terms of specific globularity and antigenicity indexes of these proteins; it is thereby hypothesized to serve as a potential basis for the differences in their immunogenic profiles [48]. PE-PGRS11 and PE_PGRS17, both cell wall associated proteins, have been shown to evoke activation and maturation of human dendritic cells along with DC-induced stimulation of CD4^+^ T-cells and enhanced pro-inflammatory cytokine responses [49]. The PGRS domain of Rv0297 encoded PE_PGRS5 leads to the production of TNF-α and IL-12 in infected macrophages [50] along with apoptosis induction [44]; the response generated was shown to be dependent on calcium ions [51]. PE_PGRS33, coded by Rv1818c, was observed to induce TNF-α; an important anti-mycobacterial cytokine, in a TLR-2 dependent manner [26]. The highly disordered nature of the PGRS domain of PE_PGRS33 enables it to aid the bacterium to enter the macrophages efficiently [52]. The PE and PGRS domains of PE_PGRS33 evoke an unusual immune response against *M.tb*; PE domain vaccinated mice splenocytes elicited cellular responses and IFN-γ production, while humoral response was induced by immunization with the PGRS domain and not by the PE domain alone of PE_PGRS33 [53]. Antibody responses against the PGRS domain of PE-PGRS33 had also been evident [54]. Higher B cell responses against PPE41, an immunologically important protein coded by Rv2430c, in comparison to PPD and Hsp10 has also been reported in TB patients [33]. *Mycobacterium smegmatis* over-expressing PE_PGRS33 and PE_PGRS26 promoted IL-10 cytokines in infected macrophages [55]. Up-regulation of pro-inflammatory cytokines, such as IL-6, IL-12p40, IL-1β and TNF-α, accompanied by pathogen survival was detected in response to PPE32 and PPE60 [38,56]. *M. smegmatis* expressing Rv1195 encoded PE13 was shown to increase IL-6 and IL-1β from infected macrophages through the p38-ERK-NF-κB host-signaling pathway [57]. Rv0335c encoded PE6, a secretory protein of *M.tb*, subdues immune responses by promoting the secretion of IL-12, IL-6 and TNF-α, most important pro-inflammatory cytokines from host macrophages in TLR4 and Myd88 dependent manner [58]. IFN-γ and IL-2 have been shown to be upregulated from polarized Th-2 cells and pro-inflammatory cytokines (TNF-α, IL-6 and IL-12p40) in response to PPE57 of *M.tb* [57,59]. PE3 was overexpressed during the chronic infection of *M.tb* and immunization with PE3 results in elevated protective immune response. [60] Bottai et al. have shown that Δ*ppe25*–*pe19* mutant of *M.tb* provided a strong attenuation and protection due to cross reactivity of the PE/PPE family of protein [20].

An anti-inflammatory response in macrophages due to the PE_PGRS30 protein in terms of the reduced production of IL-12, TNF-α and IL-6 has also been discovered [61,62]. PPE38 has been known to modulate the secretion of several other mycobacterial PE/PPE proteins and dampens host pro-inflammatory immune responses [63]. PPE18 triggers anti-inflammatory Th2 responses from macrophages by promoting IL-10 cytokines in TLR-2/MAP kinase supported pathway [64]. *M.tb* is exemplified by the suppression of pro-inflammatory cytokines (TNF-α and IL-12) for maintaining a Th-2 response to favor infection progression and eventual bacterial survival. Nair et al. demonstrated the anti-inflammatory roles of PPE18 in terms of suppressing TNF-α and IL-12p40 pro-inflammatory cytokine production by obstructing the nuclear translocation of transcription factors, such as NF-κB, c-rel, p50, and p65 [65]. PPE41, a highly polymorphic protein coded by Rv2608, has been reported with respect to its ability to induce significantly higher B-cell responses and decreased T-cellular responses [66]. PPE34 coded by Rv1917c has been shown to involve in TLR-2 dependent maturation of DCs and the subsequent secretion of very high amounts of IL-10, IL-4 and IL-5 anti-inflammatory cytokines; the production of Th-1 skewed IL-12 was not observed in response to PPE34 thereby supporting its immune evasion properties during the course of mycobacterial infection [28]. PE5 (Rv0285) and PE15 (Rv1386) modify host immune response by enhancing IL-10 and diminishing IL-12p40 cytokine levels [30]. PPE37, consisting of disordered stretches, involved in iron sequestration also leads to the secretion of high levels of IL-10 but IL-12 and TNF-α are barely detectable; thus, serving as a suitable environment for sustaining tolerant immune cells. Significant immune sero-reactivity was also observed in response to full length PPE37 protein in TB patients [45]. Another protein PE_PGRS62 was reported to exhibit induction of IL-1β and IL-6 from macrophages [67,68]. A domain-specific study exposed the preferential recognition of full-length PE_PGRS17 and PE_PGRS62 over PE domains alone [69]. Two other members of this family, PPE18 and PPE_MPTR34, were also known to modulate host responses in a TLR2-dependent manner [28,64].

*M.tb* further exploits the additional complexity of the co-operonic nature of PE/PPE protein pairs to evade the associated immune machinery by modifying Th-1 and Th-2 dependent immune balance to favor bacillary survival and the progression of disease outcomes [35,46,70,71,72]. *M.tb* PE9–PE10 heterodimers and co-operonic PE35-PPE68 interact with TLR-4 of macrophages to suppress levels of pro-inflammatory molecules IL-12 and IL-1β; upsurges that of IL-10 and monocyte chemo attractant protein-1 (MCP-1) [73]. Higher humoral response along with significant IFN-γ and TNF-α cytokine production against PPE41 and PE25/PPE41 co-operonic complex in comparison to PE25 alone has been depicted in TB patients’ sera [34]. Substantially higher levels of TNF-α production have been shown in macrophages stimulated with PE25/PPE41 complex protein; however, interestingly it did not mount IL-10 cytokine levels [34]. Mice immunized with co-operonic a PE32/PPE65 protein pair showed inhibition of Th-1 immune cytokines such as IFN-γ and IL-2 with high levels of IgG1 in serum depicting the modulation of immune responses [35]. PE/PPE proteins have also been hypothesized to act as possible exporters to augment mycobacterial virulence and pathogenesis [74].

The diverse functions of PE/PPE/PE_PGRS proteins in both subverting and stimulating immune responses are thus evident thereby hindering an appropriate cellular response required for the containment of disease [75]. Thus, further explanation of the molecular or cellular basis of such contradictory responses of these proteins belonging to same family and of almost similar structure is of utmost importance. It can be noted that the highly disordered nature of multiple stretches in these proteins (discussed below) might play an eminent role in either evoking or diminishing immune responses that augment the pathogenesis of TB.

## 4. Immune Evasion and Subversion Properties of PE_PGRS Proteins: A Possible Reflection of Antigenic Variation, Disordered Nature and Glycine Content

During the course of evolution, pathogenic bacteria developed multiple strategies to avoid or subvert host machinery, especially the mechanisms that drive protective outcomes of host immune response [76,77]. Pathogens also manipulate the outcome of the host’s immune response by altering antigen presentation pathways and engaging host immune machinery with multiple antigens [78,79]. *M.tb* utilize extremely progressive and harmonized mechanisms of immune evasion that divert or subvert the host proteins involved in neutralizing the virulence of the pathogen. In doing so, the host machinery gets engaged in evoking immune responses against the decoy antigens, thereby neutralizing the efficacy of host immune response in bacterial clearance [10,11]. Multiple PE_PGRS proteins evoke different signals that allow the pathogen to evade the host immune response [80]. PGRS domain of PE_PGRS62 protects the PE protein from ubiquitin-proteasome mediated degradation and also affects the ability of the CD8^+^ T-cells to recognize the protein, thereby conferring protection to the pathogen present within the macrophages [81].

Several pathogens employ intrinsically disordered proteins (IDPs) or disordered short stretches for a variety of moonlighting functions [82,83,84]. IDPs, by virtue of their conformational plasticity and short interaction motifs, can interact with different protein partners [85]. Such disordered effector proteins perturb host cellular cascades via favorable interactions through molecular mimicry in both viruses and bacteria [83,84,86]. The PGRS domain of PE_PGRS proteins lacks a definite three-dimensional (3D) structure and is intrinsically disordered in nature [16,44,45,87]. The transition from an ordered to a disordered state or vice versa will serve to hijack host immune machinery for subsequent survival of the pathogen [13,16,88].

The generation of antigenic variation is one of the passive mechanisms of immune evasion and subversion [89,90]. PE/PPE/PE_PGRS proteins are known to provide a major source of antigenic variations in *M.tb* and its clinical isolates [17,59]. Thus, their prospective importance in acting as a decoy antigen to the host is emphasized. The interaction of *M.tb* with macrophage offsets the Ca^2+^ signaling that causes abnormality in phagosome maturation. Ca^2+^ binds with the PE_PGRS33 and PE_PGRS61 proteins [80,91]. These calcium dependent PE_PGRS proteins decrease the Ca^2+^ concentration during the initial phase of non-specific attachment of *M.tb* with the alveolar macrophages. The decrease in the Ca^2+^ in the macrophage suppresses the phagolysosomal fusion of the *M.tb* with the acidic lysosome; thereby contributing to the survival of the *M.tb*. PE_PGRS 33 and PE_PGRS41 are cell wall associated proteins. While the PE domain of the PE_PGRS 33 is important for cellular localization, the PGRS domain of this protein is important for cellular morphology of the bacterium and its entry within the host cells. Knock-in of the *PE_PGRS33* gene in *M smegmatis* imparts endurance to the bacterium to overcome the cytotoxic effect of the macrophage and enhances the level of TNF. Although *M smegmatis* does not effectively infect host cells, recombinant strains of *M smegmatis* expressing PE_PGRS33 can colonize the lungs, spleen and liver, which is a typical feature for virulent *M tuberculosis* [88]. Ramakrishnan et al. showed that pathogenic *M.marinum* expresses two proteins (mag 24 and mag 85) that are homologous with *M.tb* PE_PGRS protein family, and are involved in granuloma formation and the replication of the pathogen within the macrophage [92]. Mice immunized with the PE domain of the PE_PGRS 33 exhibit a higher cell-mediated response while immunization with the complete PE_PGRS 33 leads to increased humoral response [53]. These studies also suggest that differential expression and the regulation of PE/PE_PGRS protein family during *M.tb* infection play a key role in enhancing the virulence features of the pathogen.

**Figure 1:**
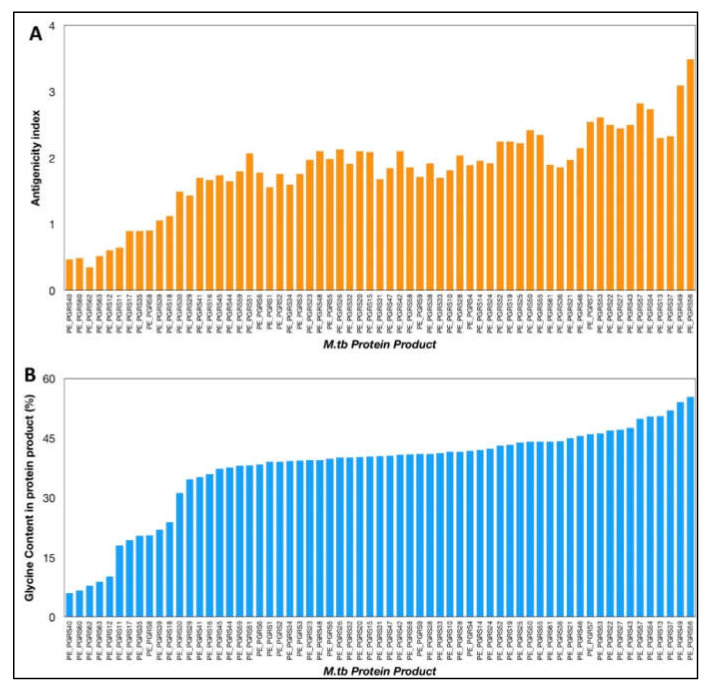
(**A**) Antigenicity index of PE_PGRS proteins of *M.tb*, as predicted by antigenicity prediction tool VaxiJen. (**B**) Glycine content of PE_PGRS proteins of *M.tb* calculated by ExpasyProtParam tool. All values were plotted in increasing order of their magnitude.

**Figure 2:**
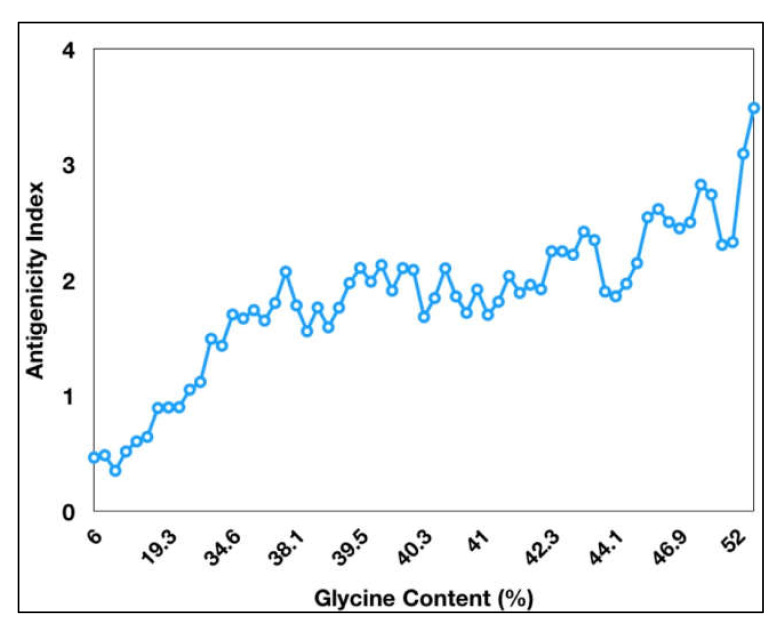
Antigenicity index of PE_PGRS proteins increases with increase in glycine content of PE_PGRS proteins. Antigenicity index was plotted against glycine percentage in linear ratio.

The VaxiJen antigenicity prediction tool shows a high antigenicity index for PE_PGRS proteins (Figure 1). The antigenicity index of PE_PGRS proteins increases as a direct function of the glycine content of these proteins (Figure 2). PGRS domain of PE_PGRS proteins was observed to be highly rich in glycine, with major chunks of Gly-Gly-Ala stretches similar to EBNA-1 antigen [81]. Glycine, a highly conserved amino acid, is known to initiate several protective and immunomodulatory responses in the host cells. Glycine modulates the function of the macrophage and evokes inflammatory cytokines, as compared to other amino acids [93]. Cell wall proteins that are rich in glycine exhibit greater antigenicity and are notable targets in several autoimmune and food borne allergies. It is important to note that the presence of high glycine content in proteins with high antigenicity indices is not just a matter of chance but points to the role of glycine-rich proteins in non-specific but targeted protective immune responses from host macrophages.

The role of PE_PGRS proteins in the immune evasion mechanism is attributed to varied and diverse patterns of the cytokine profile during *M.tb* infections [57]. While some of the PE_PGRS family of proteins, such as PE_PGRS5, PE_PGRS11, PE_PGRS17 and PE_PGRS30, evoke pro-inflammatory responses; others such as PE_PGRS26 are known to induce anti-inflammatory responses. This shows that PE_PGRS have contrasting roles in immune response and can act as a molecular switch for skewing the response as pro-host or pro-pathogen during tuberculosis [14,59]. The partial homology of PE-PGRS with EBNA domain of the Epstein–Barr virus speculates that it may play a role in the evasion of cytotoxic T-cell response to inhibit antigen processing [53,94].

Protein antigens are processed through the MHC (major histocompatibility complex) class I and MHC class II. MHC I is ubiquitously expressed on nucleated cells whereas MHC II is expressed on antigen presenting cells (APCs) including macrophages, dendritic cells, etc. Within the macrophage, *M.tb* secreted proteins are processed into smaller peptides and presented through the MHC II to the T-cells [95]. The proteins are processed through the proteasomal degradation machinery of the cell, which are translocated to the endoplasmic reticulum through the transporters associated with antigen processing (TAP) proteins [96]. CD4^+^ T cells recognize these processed antigens primed on the MHC II leading to the generation of effector and memory T-cell response against the antigenic peptides. *M.tb* involves multiple mechanisms to prevent or bypass antigen presentation processes (pathways) by inhibiting the truncation of secreted proteins into 8–25 amino acid long short peptides, required for the MHC II pathway [10,95,97]. Phagosomes, the main component of the MHC class II mediated classical antigen presentation pathway is a critical spot within the macrophages that is hijacked by the *M.tb*, resulting in inhibition of the proteasomal processing of secreted antigens. Thus, *M.tb* antigens within the macrophage are masked from being recognized by the T-cells, thereby protecting *M.tb* from cellular immune response [98]. PE/PPE/PE_PGRS proteins could be expressed as the early immunodominant antigens followed by the other functionally dominant but immuno-subdominant virulence factors. PE_PGRS proteins neutralize the effector functions of the host immune system, thereby acting as “decoy” for allowing the safe passage of other important effector molecules of the pathogen within the internal proximity of the host. Effector T cells primed against the decoy immunogen search for similar antigens throughout the cells of the host, which are discontinued by the pathogen during a subsequent phase of infection. In this way, the dominant virulent factors of *M.tb* remain unaffected by the cell-mediated immune response. The consequent subversion of T-cell response allows the bacteria to successfully establish its pathogenicity and disease progression within the host [99].

Several members of the PE_PGRS protein family were shown to induce a wide range of contradictory T-cell and B-cell responses as described in earlier sections [100]. Such responses are not specific to this protein family, rather a generalized and diverse immune profile have been observed [57]. PE_PGRS11 and PE_PGRS17 proteins are involved in the activation and maturation of human dendritic cells and boost pro-inflammatory responses [49]. The PE and PGRS domain of PE_PGRS33 evoke different immune response against *M.tb*. Mice immunized with PE domain of Rv1818c elicited cellular responses and IFN-γ production, while the humoral response was induced upon immunization with the PGRS domain and not by the PE domain alone [53]. Another study showed the generation of B-cell responses against the PGRS domain of PE_PGRS33 [54]. *M.smegmatis* over-expressing PE_PGRS33 and PE_PGRS26 show enhanced production of IL-10 cytokine levels in macrophage cell lines [55]. An anti-inflammatory response of macrophages due to the PE_PGRS30 protein in terms of the reduced production of IL-12, TNF-α and IL-6 was reported [61]. PE_PGRS33 is linked with the increased production of TNF-α and IL-10, and reduced levels of IL-12p40 [47]. In contrast, the expression of PE_PGRS16 enhances IL-12p40 levels but reduces IL-10 cytokine production [55]. The immune response generated by PE_PGRS16 was antagonistic to that of PE_PGRS26 [55,101]. These studies show that the PGRS domain plays a key role in PE_PGRS proteins and is an important target for manipulating immune response.

The elicitation of antibody responses specifically directed against the glycine and asparagine repeats has been reported [66]. PPE18 and some other 20 PE proteins have been shown to generate CD4 or CD8 mediated T-cell responses [102]. Th-2 responses and reduced IFN-Υ levels have been detected against PPE44 protein of *M.tb* [103]. PGRS domain of PE_PGRS5 protein induce TNF-α and IL-12 cytokines in macrophages [50] in a calcium dependent manner [51].

One of the most widely used anti-TB vaccine strains, BCG, is not fully capable of secreting a class of PE/PPE family proteins (specifically PE_PGRS and PPE-MPTR) due to the absence of the RD5-genetic region (containing functional Esx-5 and PPE38/71 involved in secretion) [104]. The BCG vaccine elicits a reduced repertoire of antigens during infection. In order to assess the immunogenic potential of PE/PPE/PE_PGRS proteins, Ates et al. restored the BCG strain with PPE38 locus, which improved the PE_PGRS and PPE_MPTR secretion in infected mice. Restoration of PE_PGRS and PPE_MPTR secretion neither enhanced the activation of immune cells nor boosted the protective efficacy of the restored BCG mutant strain [104]. Further studies are warranted to reveal the role of PGRS domain in improving the efficacy of recombinant BCG.

**Figure 3:**
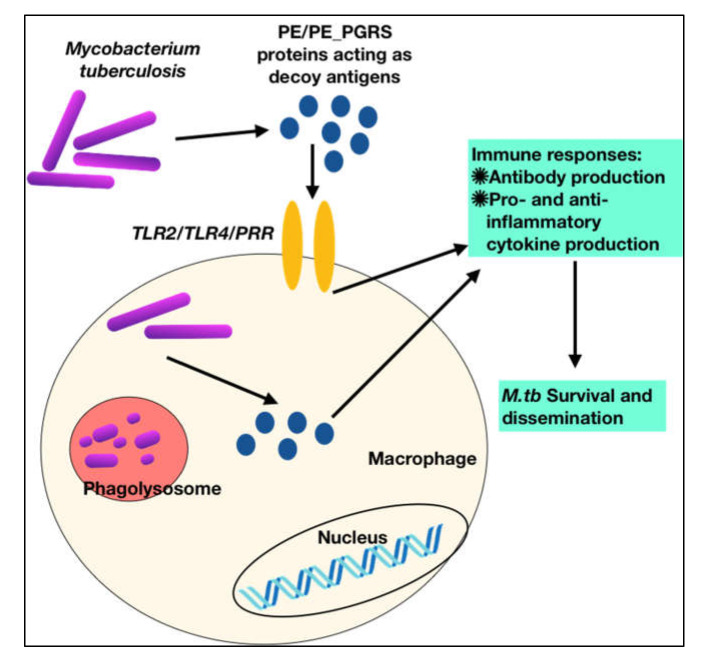
*M.tb* PPE_PGRS antigens play a role of virulent determinants by acting as an immunological decoy to capture the host immune machinery and evoke varied immune responses. This aids in evasion and subversion of host immune cellular functions during *M.tb* infection.

**Figure 4:**
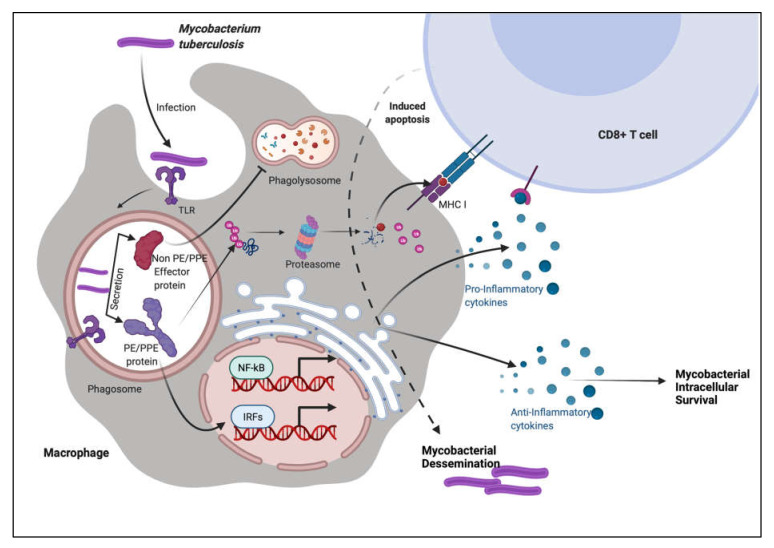
PE/PPE proteins augment the immune system of the host using decoy strategies. *M.tb* infection is most commonly found in macrophages, where the pathogen is endocytosed and transported to the endosome compartment. *M.tb* secretes non-PE/PPE and PE/PPE proteins along with other effector molecules. PE/PPE proteins are involved in the activation of immune cells. These proteins, according to the immune system, pose the greatest hazard to the cellular system. Other non-PE/PPE effectors, on the other hand, infiltrate the system and take control of the machinery, inflicting severe damage and pathogenicity.

To summarize, these observations show that PE PGRS proteins have a variety of contrasting implications, not simply the PGRS domain, which may aid in evasion and modification of immune effector activities, and hence undermine the targeting of other critical mycobacterial pathogenic proteins (Figure 3 and Figure 4). This subversion may influence the course of disease pathogenesis and lead to higher survival rates of *M.tb* within alveolar macrophages. These observations are a pointer to reconsider the immunomodulatory effects of PE/PPE/PE_PGRS proteins (Table 1, Table 2, Table 3 and Table 4), few of which are considered in vaccine formulations. Understanding the mechanisms of the PE/PE_PGRS family of proteins in evading and subverting immune responses may aid in targeting these proteins for future therapeutic interventions.

## Figures and Tables

**Table 1:** Comprehensive table showing role of different PE proteins in immune modulation of host.

Sr. No.	PE Proteins	Role in Immune Modulation	Reference
**1.**	PE17	Through JNK signaling, it regulates the transcription of pro/anti-inflammatory cytokines	[105,106]
		Increases macrophage apoptosis via chromatin remodeling in the host	
**2.**	PE6	TLR-4 agonist Pro-inflammatory cytokines are stimulated	[58,105]
**3.**	PE31	Inhibits apoptosis Pro-inflammatory cytokine production is inhibited Anti-inflammatory cytokines are stimulated	[58,107]
**4.**	PE13	Increases pro-inflammatory cytokines secretion Promotes macrophage apoptosis	[37]
**5.**	PE27	Increases pro-inflammatory cytokines secretion Contributes to Th-1-biased response	[108]
**6.**	PE11	Induces necrotic macrophage death Decreased the levels of IL-6 cytokine in macrophages	[109]
**7.**	PE5	Reduces the release of pro-inflammatory cytokines Increases the production of anti-inflammatory cytokines	[30]
**8.**	PE15	Reduces the release of pro-inflammatory cytokines Increases the production of anti-inflammatory cytokines	[30]

**Table 2:** Comprehensive table showing role of different PPE proteins in host immune modulation.

Sr. No.	PPE Proteins	Role in Immune Modulation	Reference
**1.**	PPE18	Antigen presentation by MHC class II antigens is inhibited B-cell response is inhibited	[110]
**2.**	PPE65	TLR-2 agonist Pro-inflammatory cytokines are stimulated	[111]
**3.**	PPE57	TLR-2 agonist Contributes to Th1-biased response	[112]
**4.**	PPE26	Increases the pro-inflammatory cytokines. TLR-2 agonist. Contributes to Th1-biased response.	[113]
**5.**	PPE60	Initiates macrophage pyroptosis via caspases/NLRP3/gasdermin Pro-inflammatory cytokines are stimulated TLR-2 agonist Activates Th-1/Th-17 responses in macrophages	[114,115]
**6.**	PPE11	Promotes host-cell death Pro-inflammatory cytokines are stimulated	[116]
**7.**	PPE27	Promotes host-cell death The secretion of pro-inflammatory cytokines is manipulated	[117]
**8.**	PPE44	Promotes host-cell death The secretion of pro-inflammatory cytokines is stimulated (IL-12p40 and IL-6)	[118]
			
**9.**	PPE38	Pro-inflammatory cytokines are stimulated Modulates macrophage inflammatory responses through NF-κB signaling	[63]
**10.**	PPE10	Macrophages apoptosis was regulated by reducing the expression of caspases Pro-inflammatory cytokines are stimulated	[119]
**11.**	PPE32	Through ERK1/2 signaling, it boosts the expression of IL-12p40 and IL-32 Promotes macrophage apoptosis	[38]
**12.**	PPE57	Enhances the type-I Interferon signaling pathway	[106]

**Table 3:** Comprehensive table showing role of different PE-PGRS proteins in host immune modulation.

Sr. No.	PE_PGRS Proteins	Role in Immune Modulation	Reference
**1.**	PE_PGRS41	Promotes cytotoxic host-cell death Pro-inflammatory cytokine production is inhibited	[120]
**2.**	PE_PGRS18	Modulates macrophages cytokines secretion Inhibits macrophage apoptosis	[121]
**3.**	PE_PGRS5	TLR-4 agonist ER dependent UPR activation towards stress-mediated apoptosis Pro-inflammatory cytokines are stimulated	[44,50]
**4.**	PE_PGRS11	TLR-2 agonist Pro-inflammatory cytokines are stimulated Dendritic cells are activated, which stimulate CD4^+^ T-cells	[49]
**5.**	PE_PGRS17	TLR-2 agonist Pro-inflammatory cytokines are stimulated Dendritic cells are activated, which stimulate CD4^+^ T-cells	[49]
**6.**	PE_PGRS33	TLR-2 agonist Induces the secretion of TNF-α from the macrophages	[26]
**7.**	PE_PGRS62	Latent and active TB patients shows strong antibody response	[69]

**Table 4:** Comprehensive table showing role of different PE/PPE paired proteins in host immune modulation.

Sr. No.	PE/PPE Proteins	Role in Immune Modulation	Reference
**1.**	PE32/PPE65	Inhibits pro-inflammatory cytokines Enhances anti-inflammatory cytokine Dampens Th1 response	[35]
**2.**	PE9/PE10	TLR-4 agonist Promotes apoptosis in macrophages	[72]
**3.**	PE25/PPE41	Induces necrotic macrophage death	[46]
**4.**	PE35/PPE68	Reduces the release of pro-inflammatory cytokines Increases the production of anti-inflammatory cytokines	[70]
